# Effects of mechanism of injury and patient age on outcomes in geriatric rib fracture patients

**DOI:** 10.1136/tsaco-2016-000074

**Published:** 2017-03-16

**Authors:** Helen H Shi, Micaela Esquivel, Kristan L Staudenmayer, David A Spain

**Affiliations:** 1 Case Western Reserve University School of Medicine, Cleveland, Ohio, USA; 2 Department of Surgery, Stanford University, Stanford, California, USA

**Keywords:** Geriatric, rib fractures, intensive care unit

## Abstract

**Background:**

Patients older than 65 years have 2–5 times higher mortality if they sustain ≥2 rib fractures compared to younger adults. As a result, our level I trauma center guidelines suggest that older adults with rib fractures be admitted to the intensive care unit for the first 24 hours. In this study, we evaluated the outcomes associated with these guidelines.

**Methods:**

We retrospectively reviewed all patients aged ≥65 years in our Trauma Registry who sustained rib fractures from January 2008 to March 2015. Data included demographics, comorbidities, injuries, length of intensive care and hospital stay (LOS), ventilator days, analgesic used, morbidity, mortality, and disposition.

**Results:**

97 patients aged ≥65 years with at least one rib fracture and an Abbreviated Injury Score of ≤2 for other regions were admitted. Falls caused 58% of the injuries, while motor vehicle collisions (MVC) accounted for 33%. Overall mortality was 4%. Patients who fell had a median hospital LOS that was 0.5 to 1 day longer than in those who suffered other mechanisms of injury or were involved in an MVC respectively. Patients aged ≥70 years had a median LOS of 4 days, twice that of those aged 65 to 69 years. Of the 87 patients with more than one rib fracture, 59 (68%) were not admitted directly to the intensive care unit (ICU) from the emergency department as recommended by our guidelines. 6 of these 59 patients (9%) were later transferred to the ICU and 2 of these patients expired.

**Conclusions:**

Although overall compliance with the geriatric rib fracture guideline was low, both mortality and hospital LOS were low in this group. This suggests that the guideline could be modified to reduce ICU resource usage without compromising patient outcomes.

**Level of evidence:**

Level III, retrospective cohort study.

## Background

With the aging population of the USA, there has been an increase in the number of elderly patients with trauma. Minor injuries that are typically well tolerated by younger patients can often prove fatal among the geriatric trauma population.^1^ Rib fractures are the most common type of chest trauma sustained by elderly patients and are often associated with an increased risk of complications and mortality.^2–6^ With increasing age, the ribs become more brittle and osteopenic, leading to an increased susceptibility to rib fractures in elderly patients.^7^ ^8^ Despite the seemingly minor anatomic impact of two rib fractures, they are associated with significant morbidity and mortality among geriatric patients.^1^ Kent *et al* noted that over 55% of patients older than 60 years who died of a chest injury following a car crash had no worse than rib fractures. Patients with trauma with rib fractures who were aged 65 years or older had significantly higher rates of pulmonary complications, mean number of ventilator days, length of intensive care unit (ICU) or hospital stay and mortality.^3^ ^4^ ^6^ ^9^

As a result, some trauma centers adopted clinical guidelines that involve admission to an intensive care setting in a trauma center whenever possible for elderly patients with two or more rib fractures. Usage of these guidelines can lead to significant decreases in ICU stays, hospital stays and use of mechanical ventilation.^10^ However, the effectiveness of generalization of these guidelines across different institutions has not been validated.

We conducted this retrospective case–control study to examine the implementation of this guideline and its effects on patient outcomes at Stanford Healthcare (SHC). The SHC guidelines involve ICU admission for all adults aged 65 years or older with two or more rib fractures. On admission, aggressive pulmonary hygiene and pain control strategies are implemented. We hypothesized that use of the guideline would be associated with improved outcomes in terms of length of stay (LOS) and mortality.

## Methods

This is a single-institution retrospective analysis of all patients ≥65 years of age who sustained at least one rib fracture in our Trauma Registry from January 2008 until March 2015. Patients with an Abbreviated Injury Score >2 for other regions were transferred to another hospital, and those who expired in the emergency department (ED) were excluded from the study. This was carried out to exclude confounding factors that may limit interpretation of results. The primary outcome measures were mortality, median hospital LOS and median ICU LOS.

Unadjusted analyses were performed. The Mann-Whitney U-test was used for continuous variables, and χ^2^ was used for comparison of categorical variables. Analyses were conducted using R 3.2.3 (R Foundation for Statistical Computing, Vienna, Austria). The study was approved by the Institutional Review Board at Stanford University.

## Results

During the study period, a total of 97 patients aged 65 years or older sustained rib fractures. Of these, 95 (98%) were not transferred to another hospital or did not expire in the ED. The median age of these patients was 80 years, 55% were female and 90% had two or more rib fractures (table 1). The overall mortality rate was 4%, the median hospital LOS was 4 days and the median ICU LOS was 2 days for patients whose disposition from the ED was the ICU. Of the 87 patients with more than one rib fracture, 59 (68%) were not admitted directly to the ICU from the ED as is recommended by the rib fracture trauma guidelines of our institution. Six of these 59 patients (9%) were later transferred to the ICU, and 2 of these patients expired, accounting for half of the mortality in the study.

**Table 1:** Patient demographics

	Overall (%)	1 Rib fracture	2 Rib fractures	≥3 Rib fractures
Sample size	97	10	19	68
Age	80	84.5	77	80
Gender				
Female	53 (55%)	6 (60%)	8 (42%)	39 (57%)
Male	44 (45%)	4 (40%)	11 (58%)	29 (43%)
ICU disposition	28 (29%)	0 (0%)	3 (16%)	25 (37%)
Non-ICU disposition	69 (65%)	10 (100%)	16 (84%)	43 (63%)
Transfer to ICU	6 (6%)	0 (0%)	2 (11%)	4 (6%)
Cause of injury				
Fall	57 (59%)	4 (40%)	9 (47%)	44 (65%)
MVC	32 (33%)	3 (30%)	8 (42%)	21 (31%)
Other	8 (8%)	3 (30%)	2 (11%)	3 (4%)
Median hospital LOS	4	3	3	4
Mortality	4 (4%)	0 (0%)	1 (5%)	3 (4%)

ICU, intensive care unit; LOS, length of stay; MVC, motor vehicle collisions.

For the 53 patients not admitted to the ICU, the median hospital LOS was 3 days. Forty-six (87%) of these patients were admitted to the floor, neuro-observation unit, observation unit or telemetry, and 7 (13%) of these patients were sent home without services (table 2). Of the seven patients sent home, the numbers of ribs fractured ranged from two to seven, and one patient with six fractured ribs later had an unexpected readmission. For the 28 patients who were admitted to the ICU from the ED, the median ICU LOS was 2 days, median hospital LOS was 4.5 days and mortality was 7%.

**Table 2:** ED disposition

Variable	Overall (%)	ICU—ED disposition (%)	Non-ICU—All (%)	Non-ICU –transfer to ICU (%)	Non-ICU—no transfer to ICU (%)	Non-ICU—≥2 rib fractures, no ICU transfer (%)	Non-ICU—sent home (%)
Sample size	97	28	69	6	63	53	7
Age	80	81.5	79	84.5	78	77	80
Gender							
Female	53 (55%)	15 (54%)	38 (55%)	2 (33%)	36 (57%)	30 (57%)	3 (43%)
Male	44 (45%)	13 (46%)	31 (45%)	4 (66%)	27 (43%)	23 (43%)	4 (57%)
Median ribs fractured	3.5	5	3	4	3	3	3
Median ICU LOS	0	2	0	2	0	0	0
Median hospital LOS	4	4.5	3	6.5	3	3	1
Mortality	4 (4%)	2 (7%)	2 (3%)	2 (33%)	0 (0%)	0 (0%)	0 (0%)

ED, emergency department; ICU, intensive care unit; LOS, length of stay.

Falls accounted for the injuries sustained by 59% of patients in the study while motor vehicle collisions (MVC) were the cause of 33% of patient injuries. Other causes of injury, such as bicycle accidents, pedestrian accidents, assault and motorcycle collisions, accounted for the remaining 8% of injuries (table 3). Of the 4 mortalities (4%), three were caused by falls and one by a MVC. Patients who fell had a median hospital LOS that was 1 day longer than for patients who were involved in an MVC (p=0.056) and 0.5 day longer than for patients who sustained injuries through other mechanisms (p=0.731). Additionally, patients who suffered from falls had a median of four fractured ribs compared to a median of three for patients who were involved in an MVC (p=0.951) and one for patients who suffered from other mechanisms of injury (p=0.069).

**Table 3:** Mechanism of injury

Mechanism	Sample size (% of total)	Median hospital LOS	Mortality (%)	ICU (%)	Non-ICU (%)	Transfer to ICU (%)	≥2 Rib fractures (%)
Fall	57 (59%)	4	3 (5%)	17 (30%)	40 (70%)	4 (7%)	53 (93%)
MVC	32 (33%)	3	1 (3%)	9 (28%)	23 (72%)	2 (6%)	29 (91%)
Other	8 (8%)	3.5	0 (0%)	2 (25%)	6 (75%)	0 (0%)	5 (63%)
Bicycle	4 (4%)	3	0 (0%)	2 (50%)	2 (2%)	0 (0%)	3 (75%)
Pedestrian	2 (2%)	5	0 (0%)	0 (0%)	2 (100%)	0 (0%)	1 (50%)
Assault	1 (1%)	3	0 (0%)	0 (0%)	1 (100%)	0 (0%)	0 (0%)
Motorcycle collisions	1 (1%)	7	0 (0%)	0 (0%)	1 (100%)	0 (0%)	1 (100%)

ICU, intensive care unit; LOS, length of stay; MVC, motor vehicle collisions.

**Figure 1:**
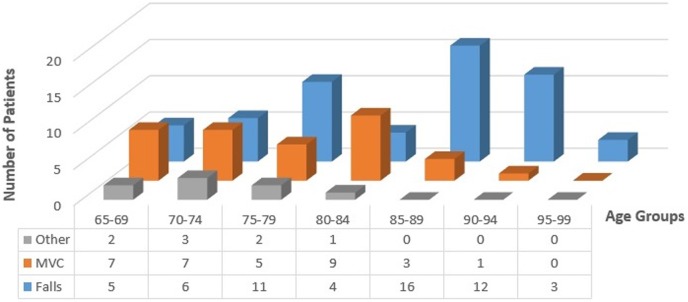
Number of patients versus age and mechanism. MVC, motor vehicle collisions.

**Figure 2:**
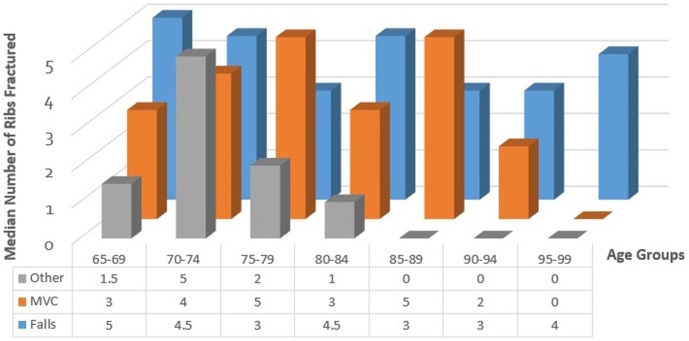
Median number of ribs fractured versus age and mechanism. MVC, motor vehicle collisions.

For patients between 65 and 75 years of age, MVC is the common mechanism of injury, accounting for 7 of 14 (50%) patient injuries in the 65–69 years age group and 7 of 16 (44%) injuries in the 70–74 years age group (figure 1). Falls became the most common mechanism of injury for patients between 75–79 and 85–99 years of age. In the 80–84 years age group, 9 of 14 patients (64%) suffered from an MVC while 4 of 14 (29%) suffered from falls. The range for the median number of ribs fractured by MVC and falls is three to five ribs across all age groups (figure 2). Interestingly, the median number of ribs fractured by falls tends to be higher for younger age groups than older age groups. The median number of ribs fractured for the age group 65–69 years is significantly higher than that for the age group 85–89 years (p=0.044).

Besides its correlation with the mechanism of injury, higher age also appears to be associated with worse patient outcomes (table 4). Patients in the 65–69 years age group had a median LOS of 2, roughly half the median LOS for those 70 years or older, which is significantly less than all other age groups except for the 80–84, 90–94 and 95–99 groups (p=0.013 for 70–74, p=0.011 for 75–79, p=0.067 for 80–84, p=0.011 for 85–89, p=0.065 for 90–94, and p=0.085 for 95–99). A significantly higher proportion of patients in the 70–74, 80–84 and 85–89 age groups were also either sent to the ICU directly from the ED or later transferred to the ICU (p=0.004 for 70–74, p=0.138 for 75–79, p=0.029 for 80–84, p=0.049 for 85–89, p=0.050 for 90–94 and p=0.201 for 95–99). Additionally, the median age of patients who are sent to the ICU from the ED or transferred to the ICU tended to be older than that of non-ICU patients (table 2), though this age difference is not significant (p=0.416 for ICU—ED disposition vs non-ICU—No Transfer to ICU; p=0.388 for non-ICU—Transfer to ICU vs non-ICU—No Transfer to ICU).

**Table 4:** Age groups

Age (years)	Sample size (% of total)	Median hospital LOS	Mortality (%)	ICU (%)	Non-ICU (%)	Transfer to ICU (%)	≥2 Rib fractures (%)
65–69	14 (14%)	2	0 (0%)	1 (7%)	13 (93%)	0	13 (93%)
70–74	16 (16%)	4	0 (0%)	7 (44%)	9 (56%)	2 (13%)	14 (88%)
75–79	18 (19%)	4	1 (6%)	5 (28%)	13 (72%)	0	17 (94%)
80–84	14 (14%)	3	0 (0%)	5 (36%)	9 (64%)	1 (7%)	13 (68%)
85–89	19 (20%)	3	1 (5%)	6 (32%)	13 (68%)	1 (5%)	15 (79%)
90–94	13 (13%)	4	2 (15%)	3 (23%)	10 (77%)	2 (15%)	12 (92%)
95–99	3 (3%)	4	0 (0%)	1 (33%)	2 (67%)	0	3 (100%)

ICU, intensive care unit; LOS, length of stay.

## Discussion

With an aging population, trauma in the elderly will continue to increase most likely with a high volume of rib fractures. Thus, it is critical to understand the best course of triage and management for this high-risk population. In the early 2000s, several studies were published showing that elderly patients with ≥2 rib fractures had higher rates of pulmonary complications, mean number of ventilator days, mean ICU LOS, mean hospital LOS and mortality, leading to the recommendation that patients aged 65 years or more with at least two rib fractures be immediately admitted to the ICU.^3^ ^8–10^ Our institutional guidelines recommend that patients ≥65 years of age with ≥2 rib fractures be admitted to the ICU for the first 24 hours to achieve adequate pain control and assistance with pulmonary hygiene. The intention behind this guideline is to admit all patients who fit this criteria to the ICU regardless of subjective evaluation of the patient's state of health. However, we found in this study that the majority of the patients who fit this criteria (68%) were not admitted directly to the ICU as per the recommendation of the guideline. Despite not following the recommendation, mortality attributable to rib fractures was 4% over the 7-year period of this study. One of these four patients had closed fracture of multiple ribs, unspecified (ICD9 807.09 2 3), another had closed fracture of two ribs (ICD9 807.02 2 3), yet another had closed fracture of five ribs (ICD9 807.05 3 3) and the last patient had closed fracture of four ribs (ICD9 807.04 3 3). All four of these patients did not have a flail chest, and their other injuries listed are external abrasions or contusions. The patient with the unspecified number of rib fractures and the one with the closed fracture of two ribs had originally been admitted to the observation unit and the floor from the ED while the patients with four and five rib fractures were admitted directly to the ICU. Six patients who were originally admitted to the floor, observation unit or telemetry subsequently required transfer to the ICU. This group of patients tended to have a lower median of fractured ribs but longer median hospital LOS compared to patients who were directly admitted to the ICU, though both had a similar median ICU LOS.

The majority of patients who were not admitted to the ICU, as recommended by the guideline, did relatively well. These patients were either admitted to the floor, neuro-observation unit, observation unit or telemetry, or sent home without services. They had a median hospital LOS of 3 days, which was significantly shorter than the 5 days for patients who were sent to the ICU at any point, although one of the patients who was sent home with six fractured ribs later had an unexpected readmission. One possible explanation for why these patients did so well may be due to improvement in the management of rib fractures in recent years. However, it is also possible that the seven patients who were sent home from the ED may have had additional complications that were not captured.

Falls were the most common mechanism of injury, especially with increasing patient age and patients who fell had a higher median hospital LOS and a higher number of ribs fractured. The relationship between falls and worse outcome measures has been associated with increased frailty in the elderly.^2^ Frailty is defined as a state of decline and vulnerability characterized by weakness and decreased physiological reserve.^1^ Older individuals tend to be frailer, making them more susceptible to more serious injury and increasing the length of their recovery time and risk of complications. Our results seem to reflect these observations, showing that ground level falls were the cause of the majority as well as the most severe injuries in the study.

Between the ages of 65 and 75 years, MVC was the most common mechanism of injury with falls accounting for 36% to 38% of the injuries in the 65–69 and 70–74 years age groups, respectively. Patients in this younger age group may be less susceptible to falling and/or to sustaining rib fractures from their falls because they are in better health or are less frail. Additionally, patients over the age of 70 years had a median hospital LOS that was 1.5–2 times longer than those of younger patients, suggesting that patients below the age of 70 years are more resilient to rib fractures. Several factors could contribute to these observations: perhaps patients are now more fit and active later into life, decreasing their frailty and much of the previous literature on this subject is several years old, so they may not reflect recent advances in geriatric and trauma care. These findings seem to suggest that perhaps not all patients aged 65 years or greater need to be admitted to the ICU for observation and perhaps that the age cut-off for the current geriatric rib fracture guidelines can be amended, as other studies have suggested that a cut-off of six rib fractures may be appropriate.^10^ However, further study needs to be carried out in order to find a more discrete age cut-off and/or number of rib fractures for ICU admission.

The limitations of our study include the small sample size, our inability to control for confounding variables and conducting our study at a single institution. Some of the variables we were unable to account for include frailty, improvements in medical technology and/or guidelines, lifestyle discrepancies, etc.

In conclusion, our study shows that mortality remains low at our institution despite limited use of the geriatric rib fracture guidelines. Additionally, we found that the median hospital LOS increases significantly for patients aged 70 years or older. With the recent advances in medicine, perhaps 70 rather than 65 years should become the new cut-off age for geriatric rib fracture guidelines. These findings suggest that further investigation into the usage of the current guideline is needed to define a discrete age cut-off and/or number of rib fractures that would benefit from ICU admission.
